# Sam Hahneman

**Published:** 1856-06

**Authors:** F. Bock


### CHARACTERISTICS OF SAM. HAHNEMANN.

Translated for the American Medical Gazette, from the German of PROFESSOR F. BOCK, by S. R. KOEHLER.

Hahnemann, as the homœopathists write, may safely be placed side by side with other great reformers, for in his love of mankind he resembles Lessing, in his acute intellect he is equal to Kant, in his activity to Baco of Verulam, and in the zeal and energey with which he overthrew the doctrines of a medicinal hiearchy, to Luther. After having practised several years—according to the story of his followers—he became disgusted with practical medicine, the exercise of which in its state at that time his conscience forbade him, and he therefore applied himself to pharmacy and chemistry for a long time, and under needy and distressing circumstances, until, while trying the cinchona bark, he discovered his genial principle, which, as he says himself, is based upon the most incontestable truth.

Quite a different thing is the biography of Hahnemann, as related by his contemporaries, and supported by undeniable facts. According to these, H. was compelled to retire from practising medicine to the translation of medical works by the want of patients, and wandered about to many different places and cities; his chemical activity he used principally for making money. Thus for instance, he sold an alkali volatile, (at Hilscher's in Leipsic,) which he pretended to have newly discovered, under the name of *Alkali Pneum*, at one Frederic's d'or the ounce, but which, by the examination of Hermbstaat, Klaproth and Karsten, (which H. never contradicted,) proved to be nothing but common borax, the ounce of which cost only a few groschens. In spite of the discovery of this swindle, H. soon made himself guilty of a second, similar one, in selling "*an infallible preventative against scarlet fever*," also at one Frederic d'or, which was nothing else but an ineffective and worthless belladonna power. In the same manner, H. showed also by his *actions in his life as a physician*, that his principal aim was money making. For not only did he *fix* his payments for the treatment of a disease, but he also *received* at least the half of the same *in advance*. Furthermore, in treating outward diseases, which, as was well known to him, could only be cured or alleviated through chirurgical aid, (for instance, hernia through trusses,) he continually sold his powders, even to poor people, at an enormous price. Even his own words throw the necessary light upon his avaricious character; for he writes in the preface to his chronic diseases: "If I was not aware for what purpose I am here upon earth—myself to become as good as possible, and to improve everything around me, so far as in my power — I would think myself very unwise, in making public an art for the general benefit, in whose possession I was alone, and which I might have made most profitable to me by keeping it secret."

Does not the charlatan, accustomed to deal in arcana, look through these words spoken with pious christian hypocrisy? What noble man and physician will think of keeping secret a beneficial mode of cure, merely for the profit?

As in monetary affairs, so also in a scientific point of view, he made himself guilty of a number of open frauds. *Thus he purposely invented and disfigured quotations*, while searching for passages in old medical writers, (Hypocrates, Bœrhave, Sydenham, De Haen, etc.,) which were to demonstrate the eternal truth of the homœopathic principles. *He further invented the effect of the cinchona bark*, upon whose fever-producing power the whole Homœpathic theory is based. For never, until now, during the often repeated experiments with the cinchona, have phenomena been produced which resemble an attack of intermittent fever, and even the Homœpathists of the present day are not able to attain this result. *The same is the case with most of the other drugs which H. pretends to have tested.* Altogether the pharmacology of H. is a mixture of inventions and contradictions, in which, probably with purpose to make investigations more difficult, all proper distinction are wanting. It is, for instance, not said of what age and constitution the person taking the medicine was, in what quantity and form it was given, how often and in what intervals it was repeated. Therefore we also do not learn anything of the development, duration, progress and end of the whole artificial disease. We can hardly trust our senses, when we read what effects are produced by every drug—even the most indifferent—upon all the organs of the body, from the crown down to the toes ; thus for instance, the quite ineffectual gold leaf can make a person become at variance with himself, discouraged, violent, cross, etc. Gross inconsequence is also apparent in H.'s experiments with medicines upon healthy persons ; for while at first he only operated with Homœopathic doses, (f. i. with the decillionth dilution of charcoal,)he will later only have large doses applied. But Hahemann reached the climax as scientific charlatan, when he suddenly undertook to prove, in a work of four volumes, that *until then the Homœopathic treatment of seven-eighths of the chronic diseases had been quite useless.* This is in open contradiction to his primary and principal maxim, and his followers, who had already for years trusted blindly in the truthfulness of their master and his mode of cure, (and who are everywhere silent about this affair,) were quite panic struck, He himself writes (in 1828) as follows: "To discover the cause why all medicines known to Homœopathy produce no true cure in chronic diseases, and if possible to get a better insight into the true character of the thousands of Chronic diseases uncured—though the Homœopathic law of cure be infallibly true—this highly important subjeet has occupied me since the years 1816, 1817, by day and night, and see! The giver of all good allowed me to solve this grand problem for the weal of mankind, gradually, by incessant thinking, untired researches, faithful observations, and accurate experiments." And what is the solution of this grand problem? It is, that seven-eighths of the chronic patients suffer from unobserved itch, one sixteenth from unobserved syphilis, and the last sixteenth from caricous tumor. Now, what shall we say of a man who, although he was quite certain that his mode of cure was of no avail against most diseases, yet for twelve years stood up for the incontestable truth of the same, and then suddenly overthrew it again!

This, then, is the man who is to be placed side by side with Luther, and to whom a monument was allowed to be erected in the intelligent City of Leipsic!!! The following facts will support what has been said before. The original of the letter copied below is to be found in the collection of autographs of a highly stationed man in Leipsic. The patient to whom the letter is addressed died in spite of the medicines prepared by Hahnemann with such "*an amount of trouble and expense*," (N. B. Shaking and dilutions.) But his two brothers-in-law are still living, and belong to a well-known family of Leipsic.

Dear Mr. N.—I send you herewith the necessary medicines, which I beg to use in the former manner. It will still become better. You promised to let me have $10 right after the holidays. But I must beg of you to remit *twenty* dollars to me to-morrow or the day after. You cannot believe what an amount of trouble and expense the preparation of *my* medicines causes me, to be able to effect with them what I really do effect, and the like no one can do but me.

Yours truly,

Dr. Sam. Hahnemann.

Two respectable citizens of Leipsic (whose names can be ascertained through the author,) gave me permission to publish the following: 1. Hahnemann's daughter, who assisted her father in Paris during his great house practice, assured Mr. N. that all the patients received only sugar plums, made of milk and sugar. 2. When Mr. N. asked a daughter of Hahnemann, who lived in the same house with him; to give him some Homœopathic remedy against his illness, he was advised by her to drink tea, as the Homœopathic medicines were nothing but dirt!

